# Copper Deficiency: An Overlooked Diagnosis

**DOI:** 10.7759/cureus.49139

**Published:** 2023-11-20

**Authors:** Hirohisa Fujikawa, Junji Haruta

**Affiliations:** 1 Center for General Medicine Education, School of Medicine, Keio University, Tokyo, JPN

**Keywords:** general internal medicine, family medicine, anemia, leukopenia, irreversible neurological damage, vitamin b12 deficiency, overlooked diagnosis, copper deficiency

## Abstract

Copper deficiency may often be overlooked due to physicians’ poor awareness of the disease. Delayed diagnosis and therapy may lead to poor prognosis of neurological function. Here, we present a 68-year-old male with an approximately one-year history of unsteady feet who had visited several clinical departments and was finally diagnosed with copper deficiency. In the present case, it took approximately one year to diagnose the condition, and the therapy of copper supplementation led to only slight improvement in subjective symptoms. Physicians should be more aware of this condition for a good prognosis of the disease.

## Introduction

Copper deficiency may be overlooked because of physicians’ poor awareness of the disease [1,2]. The time between disease onset and diagnosis can vary from months to years [3]. Copper deficiency can present with various symptoms/findings, and patients may visit multiple clinical departments, including family medicine, hematology, neurology, and orthopedic surgery [4]. Its delayed diagnosis and treatment can result in irreversible neurological deficits [4]. Here, we report a case of copper deficiency with a presentation of unsteady feet, in which delayed diagnosis and therapy led to only slight improvement in subjective symptoms.

## Case presentation

A 68-year-old man presented with a 10-month history of unsteady feet. Notably, several months earlier, during his previous physician visit, the patient underwent magnetic resonance imaging of the cervical, thoracic, and lumbar spine, which showed no remarkable findings. One month before the presentation, laboratory findings from his visit to another physician, including vitamin B12 and folic acid levels, were also unremarkable. The patient denied any past medical history of neurological disorders. He reported drinking no alcohol. He denied any history of drug abuse. There was no family history of note.

Romberg’s sign was positive upon examination. However, other neurological findings were unremarkable. Laboratory tests showed a white cell count of 4.0 $\times$ 10^9^/L (normal range, 3.5-9.1 $\times$ 10^9^/L), which was just above the lower normal limit, a hemoglobin level of 129 g/L (normal range, 135-170 g/L), and a mean corpuscular volume of 93.2 fL, indicating mild normocytic anemia. Other results, including liver and kidney function tests, were within normal limits.

Considering the mildly decreased white blood cell and hemoglobin levels, the probable presence of dorsal column disorder, and normal vitamin B12 and folic acid levels, we suspected the patient had copper deficiency. Additional laboratory examinations revealed low serum copper and ceruloplasmin concentrations of 64 $\mu$g/dL (normal range, 70-133 $\mu$g/dL) and 16 mg/dL (normal range, 21-37 mg/dL), respectively. Ultimately, the patient was diagnosed with copper deficiency. However, no obvious cause of copper deficiency (history of gastrointestinal surgery and using zinc-containing supplements and alcohol) was identified.

To treat copper deficiency, we advised the patient to take two to three cups of pure cocoa daily (0.4-0.6 mg of copper per day) because there are no medications or foods containing copper alone in Japan. Two months after he began drinking cocoa, although the level of copper in the serum improved to the normal range, the subjective symptoms that bothered him had not changed significantly. In contrast, his objective findings had improved; his white cell count and hemoglobin level increased to 5.2 $\times$ 10^9^/L and 141 g/L, respectively. The Romberg’s sign was negative, although there was still a slight rocking of the body with the eyes closed in the standing position. The patient has continued to consume cocoa since then.

## Discussion

In the present case, a patient with an almost one-year history of foot unsteadiness was diagnosed with copper deficiency. This case report has two significant findings. First, patients with copper deficiency may present with various symptoms/findings. Second, copper deficiency may frequently be overlooked, and neurological symptoms may become irreversible or only partially improve if treatment is delayed.

Copper is an essential trace element in the human body. It is absorbed in the stomach, duodenum, and jejunum and spreads throughout the body through the liver. It acts as a prosthetic group in key enzymes and is crucial in the bone marrow and neurological system [5]. In their largest case series, Jaiser et al. showed that the etiology of copper deficiency included previous upper gastrointestinal surgery in 47% of the cases, zinc overload in 16% of the cases, and malabsorption in 15% of the cases [5]. Prolonged parenteral nutrition and alcohol abuse can induce copper deficiency [2]; however, the cause of copper deficiency remains undetermined in a significant percentage of patients (20%) [5]. In the present case, the patient had no history of gastric surgery. There was no history of suspected malabsorption (e.g., celiac disease and inflammatory bowel disease). A thorough history-taking was conducted, and he denied ingesting alcohol or zinc supplements. Therefore, we diagnosed the patient with idiopathic copper deficiency, but we will continue to look for hidden causes of the condition.

Clinical manifestations of copper deficiency are mainly hematological and neurological [1]. Hematological manifestations include leukopenia and various types of anemia (microcytic, normocytic, or macrocytic) [1,5]; however, thrombocytopenia is relatively uncommon [5]. Bone marrow findings of patients with copper deficiency can mimic myelodysplastic syndrome [6,7]. Neurologically, it can present as myelopathy and peripheral neuropathy simulating subacute combined degeneration [5,8]. Because of the wide range of clinical manifestations of copper deficiency, the differential diagnosis is diverse, including vitamin B12 deficiency, folate deficiency, drug toxicity, infection, autoimmunity, myelodysplastic syndrome, aplastic anemia, and lymphoma with bone marrow involvement [9]. Of these, it is difficult but essential to differentiate copper from vitamin B12 deficiency because the phenotypes are very similar [2]. Patients with copper deficiency, similar to those with vitamin B12 deficiency, present with various symptoms and may see physicians of various departments.

Copper supplementation leads to prompt and full recovery of blood cell abnormalities [5,10]. In contrast, as the present case indicated, the neurological symptoms will become irreversible or improve only partially if treatment is delayed [5]. The improvement in neurological signs and symptoms is variable, and most patients have some degree of residual disability [3,11]. Therefore, it is crucial to diagnose and treat copper deficiency early in patients [2,5]. Diagnosing it depends on the laboratory examinations of copper and ceruloplasmin [5].

There are several therapeutic strategies for copper deficiency. In general, oral supplementation with 2 mg of copper a day, which is twice the recommended intake (0.9 mg/day) [12], is recommended. Conversely, in patients with copper deficiency, if there are no intestinal problems, absorption efficiency from the intestinal tract is increased [13], so an extreme increase may not be necessary. In this case, we chose two to three cups of pure cocoa per day (0.4-0.6 mg of copper per day) with reference to a previous case report [2]. While there have been several studies on the therapeutic effect of cocoa powder on patients with copper deficiency due to long-term enteral nutrition [14,15], the efficacy of oral cocoa intake in those with other causes has not been determined, except for a few case reports [2,16]. Our case is significant in contributing to the proof of its efficacy.

## Conclusions

Copper deficiency may often be overlooked. However, as the present case indicated, delayed diagnosis and therapy may result in poor prognosis. Therefore, physicians should be more aware of this condition because correct diagnosis and timely treatment may enhance patient outcomes.
